# *In vivo* and *in vitro* Digestibility of an Extruded Complete Dog Food Containing Black Soldier Fly (*Hermetia illucens*) Larvae Meal as Protein Source

**DOI:** 10.3389/fvets.2021.653411

**Published:** 2021-06-11

**Authors:** Livio Penazzi, Achille Schiavone, Natalia Russo, Joana Nery, Emanuela Valle, Josefa Madrid, Silvia Martinez, Fuensanta Hernandez, Elena Pagani, Ugo Ala, Liviana Prola

**Affiliations:** ^1^Department of Veterinary Sciences, University of Turin, Grugliasco, Italy; ^2^Faculty of Veterinary Medicine, University of Teramo, Teramo, Italy; ^3^Department of Animal Production, University of Murcia, Murcia, Spain

**Keywords:** sustainability, pet food, digestibility, protein, novel feed materials, insect meal

## Abstract

Growing attention is being directed toward insects as a novel and sustainable source of protein for pet food. The aim of the study was to evaluate nutrient digestibility of a diet containing black soldier fly larvae as its main protein source. Moreover, the purpose of the study was to compare the traditional *in vivo* total collection method with the *in vivo* marker method and *in vitro* digestibility method. Two isonitrogenous and isoenergetic dry diets containing either venison meal (CTRL diet) or black soldier fly larvae meal (BSF diet) as their primary sources of proteins were fed to six adult dogs, according to a Latin square design. The digestibility of nutrients was determined using both *in vivo* (“total collection” and “internal marker” approaches) and *in vitro* methods. The two diets showed similar nutrient digestibility values for dry matter, organic matter, ether extract, ash, and phosphorus. However, a statistical trend (*p* = 0.066) was observed indicating greater protein digestibility in the BSF diet compared with the CTRL diet. Calcium digestibility was higher in the BSF diet compared with the CTRL diet (*p* = 0.018). On the contrary, fiber digestibility was lower in the insect-based diet compared with the venison diet (*p* < 0.001). There was no difference between total collection and internal marker methods in the assessment of *in vivo* digestibility for any of the nutrients considered. The *in vitro* digestibility values for dry matter, organic matter, and crude protein, as well as the estimated *in vivo* digestibility of organic matter and crude protein by the means of the predictive equation, were aligned with the *in vivo* results, although *in vitro* estimations were consistently higher compared with those obtained by *in vivo* analysis. Digestibility analysis of a dog food containing insect meal as the sole source of protein (36.5% inclusion) showed promising results in terms of it presenting similar values as a meat-based diet, indicating its suitability as a sustainable protein source for pet food. Moreover, the study showed that both the *in vivo* marker method and the *in vitro* method could be possible alternatives to the traditional total collection method in digestibility trials.

## Introduction

With the livestock industry at its limit in terms of sustainable production capacity, and the pet food business in constant growth, new sources of protein are being sought in order to meet the market's demand and the expectations of pet owners (1). Insects may provide a possible solution as an alternative feed, since they can partially replace traditional feed sources, while they also provide a means to bio-converting organic waste (2). Of the various insects being considered, the black soldier fly (*Hermetia illucens*) is showing particular promise due to its immediate potential for large-scale production (3).

The black soldier fly (BSF) has a balanced protein composition and one of the highest amino acid scores compared with other currently reared insects or traditional protein sources (such as fish meal) (4). Compared with crickets and mealworms, BSF boasts a more stable nitrogen and phosphorus composition and has a more advantageous feed conversion ratio (5). It can also be considered a possible sustainable solution due to the possibility of rearing the insects on materials deemed unsuitable for human nutrition, such as alimentary by-products and organic substrates (6).

As pointed out by Böhm et al. (7), insects may constitute an appropriate novel protein source for dogs, presenting cutaneous adverse food reactions. Nevertheless, societal negative opinions about the use of insect meal in pet nutrition have arisen, especially due to insect phobia and concerns about safety. Security aspects about insect consumption were also discussed critically in EFSA Scientific Opinion (8), where uncertainty regarding the risk of non-processed items, due to the lack of data, has been acknowledged. However, EFSA concluded that microbiological risks are expected to be comparable with other food raw materials, provided that insects are fed with allowed feedstuff. Consumers from Western countries still continue to have prejudices regarding the introduction of insects in their diet (9), and, due to the current “humanization trend” (10), this fact could be also translated to their pets. Notwithstanding, public opinion seems to be less concerned about the use of veterinary-prescribed diets based on insects (11). Indeed, veterinarians have expressed interest in hypoallergenic food alternatives prepared using insects (12). According to the Commission Regulation (EU) 2020/354 (March 4, 2020) (13), a product can be claimed to reduce ingredient and nutrient intolerances if it is composed of hydrolyzed proteins or selected and limited protein sources or selected carbohydrate sources. Therefore, according to the current European Regulations, a product composed only of insects as the main source of protein could be considered with the particular purpose of reduction of food intolerance. Concurrently, and reflecting the growing interest in this field of research (14), various recent studies have investigated the possibility of feeding BSF larvae to poultry (15–18), fish (19–21), and swine (22, 23). Recently, a thorough review from Bosch and Swanson (24) explored in depth the palatability, digestibility, and nutritional aspects of the inclusion of insects in dog and cat diet, showing the potential of insects as future pet food products.

The aim of the present study was to evaluate the inclusion of defatted BSF larvae meal in extruded dog food in terms of its *in vivo* and *in vitro* digestibility, in order to assess its suitability for the pet food market. Furthermore, the purpose of the study was to evaluate if the *in vivo* marker method and the *in vitro* digestibility method could be comparable to the traditional *in vivo* total collection method also in these particular diets. The estimated *in vivo* digestibility of organic matter and crude protein calculated by means of predictive equations utilizing data obtained by *in vitro* analysis was also assessed.

## Materials and Methods

All the experimental procedures were approved by the Bioethics Committee of the University of Turin (Italy) (prot. n. 336595).

### Animals and Experimental Design

Six clinically healthy West Highland White Terrier adult dogs [three males and three females, 3 ± 1.8 years old, 7.2 ± 0.8 kg BW, BCS ranging between 4.5 and 5.5 on a nine-point scale (25)] were fed two isonitrogenous and isoenergetic dry extruded diets (control vs. insect diet) according to a Latin square design. During the digestibility experiment, the dogs were housed individually in 3 ×3-m kennels and had *ad libitum* access to fresh water. The dogs were allowed to walk freely for 1 h per day in a concrete outside the pen and play with toys during the adaptation periods.

### Diets and Digestibility Protocol

Two diets were tested during the trial. The diets were formulated to be isoenergetic and isonitrogenous. In the control diet (CTRL diet), the protein source was provided in the form of processed [rendering process, method III, according to the EU Reg. 142/2011 (26)] deer (*Cervus elaphus*) protein, whereas the insect diet (BSF diet) provided defatted BSF (*H. illucens*) larvae meal as its sole protein source (Hermetia Futtermittel GbR, Baruth/Mark, Germany). The chemical composition, amino acidic profile, and ingredient composition of both diets are shown in Table 1. Diets were formulated and balanced in order to meet nutrient requirements in accordance with the FEDIAF (27) nutrient guidelines for dogs.

**Table 1:** Ingredients and nutritional composition of the experimental diets.

	**CTRL^a^**		**BSF^b^**	
**Ingredients**	**(% as fed)**	**(% of DM)**	**(% as fed)**	**(% of DM)**
Potato meal	51.5		54	
Venison meal	40		-	
Black soldier fly meal	-		36.5	
Vitamin and mineral premix	3		3	
Oils and fats^c^	2.5		2	
Yeast (hydrolysate)	2		2	
Calcium carbonate	-		1.5	
Other ingredients^d^	1		1	
**Nutrient and chemical composition^e^**				
Dry matter	93.80	-	96.04	-
Organic matter	86.11	91.80	90.21	93.93
Crude protein	16.97	18.09	20.70	21.55
Ether extract	17.42	18.57	15.61	16.25
Crude fiber	5.77	6.15	4.09	4.26
Ash	7.69	8.20	5.83	6.07
Calcium	1.03	1.10	0.87	0.91
Phosphorus	0.93	0.99	0.53	0.55
Collagen	2.72	2.90	0.88	0.92
Hydroxyproline	0.34	0.36	0.11	0.11
**Amino acidic profile^e^**				
Aspartic acid		1.88		2.09
Serine		0.68		0.79
Glutamic acid		1.98		2.19
Glycine		1.14		1.01
Histidine		0.31		0.49
Arginine		0.86		1.02
Threonine		0.60		0.68
Alanine		0.87		1.15
Proline		1.12		1.07
Cysteine		0.15		0.16
Tyrosine		0.40		0.78
Valine		0.71		1.01
Methionine		0.23		0.39
Lysine		0.80		0.97
Isoleucine		0.53		0.69
Leucine		1.03		1.23
Phenylalanine		0.64		0.79
ME (MJ/kg)^f^	15.66		16.44	

a *CTRL, control diet*;

b *BSF, black soldier fly diet*;

c *Poultry purified fat, sunflower oil*;

d *Digest (hydrolyzed poultry liver), mineral, and vitamin pre-mix*;

e *Analyzed*;

f *Estimated according to FEDIAF (27)*.

Venison was chosen as the primary protein source for this trial since it is one of the protein sources usually incorporated in commercial foods for dogs which show adverse food reactions; similarly, insect meal showed a similar potential (7). Nevertheless, venison meal is more expensive than other common sources of proteins as well as insect meal so far and, for these reasons, was deemed eligible for the comparison of the diets.

The trial was conducted according to the guidelines of Carciofi et al. (28) regarding the use of a marker method and the total collection method for assessing *in vivo* total tract apparent digestibility. Chromium oxide (Cr_2_O_3_) was used as digestibility marker. It was added to a final concentration of 2.5 g/kg of diet. A 5-day test diet adaptation period preceded 5 days of feces collection during the experimental trial.

Food was weighed each day, divided into two equal portions, and given to the animals at 9 a.m. and 5 p.m. in stainless-steel bowls. Food quantity was administered considering maintenance energy requirements according to the FEDIAF equation (110 kcal × BW^0.75^) (27). Bowls were removed before the next meal, and any uneaten food was weighed and recorded. Feces were collected twice daily, weighed, and kept frozen at −20°C until analysis.

### Chemical Analyses

At the end of the collection period, pooled individual feces were thawed, homogenized, and freeze-dried. Feces samples were freeze-dried using a laboratory freeze dryer (5Pascal, Trezzano sul Naviglio, Italy). The process of lyophilization consisted of dry sublimation with water evaporation under low pressure (0.200 mbar) until the samples reached room temperature (25°C). Both the foods and freeze-dried feces were ground to pass through a 1-mm sieve and stored in airtight plastic containers for laboratory tests. The dry matter (DM) of the foods was determined by drying the samples at 103°C to constant weight. The foods and feces were analyzed according to the AOAC (29) standard procedures; thus, ash was determined by muffle furnace incineration (section 942.05), crude protein (CP) was ascertained using the Kjeldahl method (section 954.01), and ether extract (EE) was analyzed following acid hydrolysis (section 954.02). In addition, diet crude fiber (CF) was determined using the method described in section 962.09 (29), and amino acid content by HPLC (Waters Alliance System with a Waters 1525 Binary HPLC Pump, Waters 2707 Autosampler, and Waters 2475 Multi λ Fluorescence Detector, Milford, USA) after pre-column derivatization (30) in samples ground to pass a 0.5-mm sieve. The detection limit ranged from 2.9 to 20.1 pmol/μl depending on the amino acid. Tryptophan was not analyzed.

Samples of foods and feces were burnt to ashes and acid-digested in the microwave (31), prior to the determination of chromium concentrate by inductively coupled plasma optical emission spectrometry (ICP-OES). Calcium and phosphorus were also determined by ICP-OES in the absence of the previous incineration.

Hydroxyproline and the related collagen content were assessed according to the colorimetric method adapted by Kolar (32) and described in the AOAC (29) section 990.26. The acid hydrolysis of the sample was performed under heat; an oxidizing agent was added to the sample, and oxidized hydroxyproline was measured photometrically.

### *In vivo* Digestibility Calculations

Apparent total tract digestibility coefficients (ATTDC) of the individual dietary elements of the two diets were calculated as follows:

a) Total fecal collection method (TFC):

$$\begin{array}{l} \text{ATTDC }{\text{X}}_{\text{diet}}\;\left(\%\right)\;=\;\left[\left(\text{total }{\text{X}}_{\text{diet}}-\text{total}{\text{X}}_{\text{feces}}\right)/\text{total }{\text{X}}_{\text{diet}}\right]\;\times \;100 \end{array}$$

where X is the total contents of DM, organic matter (OM), CP, EE, ash, calcium, or phosphorus in the consumed food or feces produced (X_diet_ and X_feces_, respectively);

b) Marker method (Cr_2_O_3_):

$$\begin{array}{l} \text{ATTDC }{\text{X}}_{\text{diet}}\;\left(\%\right)=\{[{(\text{X}/\text{C}{\text{r}}_{2}{\text{O}}_{3})}_{\text{diet}}\\ -{\left(\text{X}/\text{C}{\text{r}}_{2}{\text{O}}_{3}\right)}_{\text{feces}}]/{\left(\text{X}/\text{C}{\text{r}}_{2}{\text{O}}_{3}\right)}_{\text{diet}}\}\times 100 \end{array}$$

where X represents the concentrations of DM, OM, CP, EE, ash, calcium, or phosphorus in the diet or feces;

Cr_2_O_3_ represents the chromium oxide concentration in the diet or feces;

(X/Cr_2_O_3_)_diet_ = ratio between nutrient (X) and Cr_2_O_3_ concentration in the diet;

(X/Cr_2_O_3_)_feces_ = ratio between nutrient (X) and Cr_2_O_3_ concentration in the feces.

### *In vitro* Digestibility

The *in vitro* digestibility of DM, CP, and OM of the food was determined (in triplets) employing the methods described by Hervera et al. (33, 34). The methods involve two phases: the first entails incubation for 2 h under conditions simulating gastric digestion (pH 2, 39°C, and inclusion of pepsin), whereas the second phase simulates 4 h of post-gastric digestion (pH 6.8, 39°C, and inclusion of a pancreatin preparation for enzymatic digestion). The resulting residue was filtered, dried, and weighed to determine the remaining DM content and incinerated to determine the residual OM content. Residual CP was determined by ascertaining the nitrogen content of the residue (using the Kjeldahl method) and considering a N:P conversion factor of 6.25. The *in vitro* digestibility of DM, OM, and CP was calculated as the difference between the amount of each initial nutrient in the sample vs. the undigested residue, divided by the initial nutrient content of the sample.

### Estimated Digestibility

Data from the *in vitro* digestibility analyses were also used to estimate *in vivo* OM and CP digestibility according to the regression equations reported by Hervera et al. (33, 34):

Estimated digestibility of OM (%) = −9.15 + 1.06 × *in vitro* OM digestibility (%) (33);
Estimated digestibility of CP (%) = 37.91 + 0.52 × *in vitro* CP digestibility (%) (34).

### Statistical Analysis

The statistical unit was the individual dog for *in vivo* digestibility trials, and the diet for *in vitro* digestibility trials. The comparisons between diets (CTRL vs. BSF) and methods (*in vivo* TFC vs. Cr_2_O_3_) were analyzed using two-way ANOVA, considering the diet (D) and the method (M) of *in vivo* digestibility calculation as the source of variation, respectively. Before testing for group and method differences, the normality of the data distribution and the homogeneity of variance were assessed by the means of the Shapiro–Wilk test and Levene test, respectively. The significance level was set at *p* = 0.05. A statistical trend was considered for *p* ≤ 0.10. All statistical analyses were performed using R Software (version 3.6.1) (35).

## Results

The foods were well-accepted during all the trial lengths, and no episode of nausea or vomiting has been reported. The *in vivo* ATTDC digestibility results are summarized in Table 2. The two methods used to estimate *in vivo* digestibility (TFC and Cr_2_O_3_) showed similar results between the CTRL and BSF groups in relation to DM, OM, EE, ash, and phosphorus. The ATTDC of CF was significantly lower (*p* < 0.001) in the BSF diet compared with the CTRL diet. On the contrary, the ATTDC of calcium was significantly higher (*p* < 0.05) in the BSF compared with the CTRL diet. A statistical trend (*p* = 0.066) was observed for the ATTDC of CP, being higher in the animals fed the BSF compared with the CTRL diet.

**Table 2:** Comparison of the *in vivo* digestibility using the total fecal collection method (TFC) and *in vivo* digestibility with marker (Cr_2_O_3_) in six dogs (mean values are presented).

	**TFC^a^**		**C_2_O_3_**		**SEM**	***p*****-value**		
	**CTRL^b^**	**BSF^c^**	**CTRL^b^**	**BSF^c^**		**D^d^**	**M^e^**	**D × M^f^**
***In vivo*** **digestibility (%)**								
Dry matter	82.11	82.17	83.05	83.83	0.52	0.698	0.241	0.740
Organic matter	86.23	85.04	86.98	86.46	0.45	0.358	0.247	0.719
Crude protein	72.41	75.80	74.04	78.22	1.01	0.066	0.311	0.842
Ether extract	96.58	96.40	96.72	96.75	0.14	0.800	0.411	0.717
Crude fiber	43.13	18.83	45.78	23.60	3.18	<0.001	0.393	0.798
Ash	32.73	35.76	35.88	41.39	1.95	0.292	0.280	0.757
Calcium	12.16	24.88	19.19	31.62	2.61	0.018	0.162	0.976
Phosphorus	20.77	21.46	26.17	25.83	2.00	0.946	0.280	0.908

a *TFC, total fecal collection*;

b *CTRL, control diet*;

c *BSF, black soldier fly diet*;

d *D, diet*;

e *M, method*;

f *D × M, diets and method interaction*.

No statistical differences were observed between the two ATTDC methods (TFC vs. Cr_2_O_3_). Furthermore, no statistical interaction between diets and methods was found.

The *in vitro* digestibility data and estimated *in vivo* digestibility results, obtained utilizing the regression equations described in Hervera et al. (33, 34), are reported in Table 3. The digestibility values for DM, OM, and CP obtained using the *in vitro* method were higher for both the CTRL and the BSF diet (by an average of +8.43, +5.25, and +6.08%, respectively) compared with those obtained using *in vivo* methods. The estimations of *in vivo* digestibility of OM and CP (based on *in vitro* data) were consistently higher than the data obtained using *in vivo* ATTDC methods: *in vitro* estimation of *in vivo* digestibility overestimated OM and CP digestibility by up to 4.0% and 9.8%, respectively, compared with the *in vivo* methods.

**Table 3:** Comparison of the *in vitro* digestibility of the two diets (CTRL vs. BSF) and estimated *in vivo* digestibility based on the *in vitro* results.

	**CTRL^a^**	**BSF^b^**
***In vitro*** **digestibility (%)**		
Dry matter	90.65	91.79
Organic matter	90.82	92.04
Crude protein	80.06	82.33
**Estimated** ***in vivo*** **digestibility (%) based on the** ***vitro*** **results**		
Organic matter^c^	87.12	88.41
Crude protein^d^	79.54	80.72

a *CTRL, control diet*;

b *BSF, Black soldier fly diet*;

c *According to Hervera et al. (33) for OM estimation*;

d *According to Hervera et al. (34) for CP estimation*.

## Discussion

This study evaluated the nutritional quality of defatted BSF larvae meal as a potential sustainable novel raw material for pet food, to be integrated into extruded diets as a protein source. In addition, it explored the suitability of the *in vivo* marker method and the *in vitro* digestibility method with the traditional *in vivo* total collection method.

Although the control (containing venison meal) and insect-based diets were formulated to be isonitrogenous, our analysis showed CP content to be almost 4% lower in the former (16.97 vs. 20.70%, respectively); the discrepancy between the diets was nevertheless within the limits stipulated in the EU regulation 2017/2279 regarding “Tolerances for analytical constituents” (36). It is also important to remember that since chitin is a nitrogen-containing polysaccharide, this could also have led to a mild overestimation of the protein content in the BSF diet (6, 37).

We must also acknowledge that the higher crude protein content of the BSF diet compared with the CTRL diet could be an overestimation due to our use of a nitrogen to protein (N:P) conversion factor of 6.25. In fact, several authors recently pointed out that this conventionally used conversion factor may lead to the overestimation of protein content in a variety of feedstuffs (38, 39), including insect meals (40, 41). Furthermore, although Finke et al. (42) estimated that the amount of nitrogen in insect chitin would not significantly affect the total amount of nitrogen, other authors support the hypothesis that the presence of non-protein nitrogen (NPN) in insect CP could cause the overestimation of CP (40, 41).

In our trial, the ATTDC of DM, OM, and EE were similar in both BSF and CTRL groups, whereas the ATTDC of CP were higher in the BSF vs. CTRL group. A similar result was obtained by Lei et al. (43), where increasing levels of BSF meal inclusion (at 0, 1, and 2%) in Beagle dog rations raised nitrogen digestibility, whereas EE digestibility remained similar to that of the control diet. However, Gariglio et al. (18) observed that up to 9% BSF meal inclusion in the diet of growing Muscovy ducks did not change diet digestibility, with the exception of the ATTDC of EE, which was improved in BSF groups. In line with these data, Biasato et al. (23) observed no change in the ATTDC of BSF diets (up to 10% inclusion) in growing piglets. Similarly, Freel et al. (44) did not notice any difference in ATTDC of DM, CP, and EE in a trial involving 56 Beagle dogs fed with diets containing graded levels of BSF meal (5.0, 10.0, and 20.0%) and BSF oil (1.0, 2.5, 5.0%). Furthermore, in a study where BSF meal completely replaced soybean meal in the diet of laying hens, Cutrignelli et al. (45) found BSF to correlate with lower crude protein digestibility, whereas lipid digestibility remained unaffected. Likewise, Kröger et al. (46), in a study involving 12 Beagles, observed a decrease in ATTDC of CP in the BSF group compared to the control group, while the ATTDC of DM was increased when dogs were fed the diet containing the BSF meal (at 20.0% of inclusion). This result could be explained by differing levels of chitin, which can negatively affect protein digestibility (47). Indeed, the reported difference in fiber digestibility between the diets supports this result and explanation, since chitin gets recognized as part of the crude fiber fraction during the analysis (48). Furthermore, the mean values of crude protein ATTDC (for BSF-based diets) observed in our study were in line with those found in Kröger et al. (46) but below those recovered in Freel et al. (44).

Hydroxyproline can be used as an index of protein quality (49), due to its being a marker of collagen content (50). The levels of collagen and of hydroxyproline were higher in the control diet compared with the BSF diet, probably due to the fact that collagen is limited in insect meal compared to that in vertebrate protein meal. This could also explain the higher level of digestibility of the BSF diet compared with the control diet, at least with regard to crude protein digestibility, since the net protein utilization of collagen is zero (51). Collagen content also influences the N:P ratio of protein sources, and consequently the real CP content of the diets, in particular that of the control diet (39). It may also be speculated that the control diet had a decreased crude protein digestibility due to the higher ash content; however, high levels of crude ash did not appear to decrease protein digestibility, as previously reported by Bockskopf and Kamphues (52).

The difference in calcium digestibility could be due to the use of different ingredients to adjust the calcium level of the diets. Indeed, calcium carbonate was added to the BSF diet to obtain the minimum requirements for dogs, whereas in the CTRL diet the calcium requirements were satisfied by the presence of ground bone in the venison meal (thus avoiding the need for any calcium salt addition), and this could have led to the discrepancy. Interestingly, Lei et al. (43) noticed significant increases in the level of calcium in the blood of beagles as the BSF larvae meal content of their food was increased. This result points toward a potential increase in the bioavailability of this macro-element that depends on the inclusion of BSF larvae meal in the diet; however, further investigations are required to confirm and understand the basis of any possible relationship.

It is important to note that no statistical differences were observed between the ATTDC values determined using the marker method and the total collection method for both CTRL and BSF diets, confirming the validity of the marker method as an alternative to the total collection method (28). The values of *in vitro* DM, OM, and CP digestibility were also similar to the results obtained with the two *in vivo* methods, despite being, in line with the previous literature (33, 34), slightly overestimated in the former. We also evaluated whether the equations for the estimation of *in vivo* crude protein and OM digestibility, utilizing *in vitro* digestibility data, as described in Hervera et al. (33, 34), fitted with the results obtained in this study (shown in Table 3). Since the predictive equations proposed were only used to assess feedstuff based on vertebrates and, to our knowledge, no other study inspected if they could be applicable to invertebrates, we decided to include these findings. For both the venison and insect diet, the predictive equations gave slightly overestimated values compared with the mean of the *in vivo* digestibility results, even though they were substantially similar from a nutritional perspective. Indeed, the discrepancy between the crude protein digestibility estimated using the equation and the *in vivo* crude protein digestibility results ranged from 3.2 to 9.8%, whereas the overestimation of the OM digestibility ranged from 0.2 to 4.0%, with lower deviations and a narrower range. According to these results, predictive equations utilizing *in vitro* digestibility values appear to constitute a valid tool for the analysis of feedstuff digestibility and therefore offer a means to reduce, if not avoid, the use of live animals.

## Conclusions

The present study suggests that the inclusion of BSF in extruded diets for dogs (at 36.5%) offers a promising alternative source of dietary protein for this species, in particular in relation to the digestibility profile of crude protein, crude fat, and OM. Our findings also highlight the need for further studies in order to understand the effect of chitin on fiber digestibility and mineral absorption in a BSF-based diet.

## Data Availability Statement

The raw data supporting the conclusions of this article will be made available by the authors, without undue reservation.

## Ethics Statement

The animal study was reviewed and approved by Ethic Committee of Turin University. Written informed consent was obtained from the owners for the participation of their animals in this study.

## Author Contributions

AS, EP, LPe, and LPr conceived and designed the experiment. EP and NR collected the experimental data. EV, FH, JM, JN, and SM carried out the chemical analyses. AS, LPe, and UA performed the statistical analysis. All the authors interpreted the data. AS, LPe, and LPr wrote the first draft of the manuscript. All the authors reviewed the manuscript for intellectual content and gave approval for the final version to be published.

## Conflict of Interest

The authors declare that the research was conducted in the absence of any commercial or financial relationships that could be construed as a potential conflict of interest.

## Abbreviations

CTRL diet: venison meal-based diet/control diet
BSF diet: black soldier fly larvae-based diet/insect diet
BSF: black soldier fly
ME: metabolizable energy
DM: dry matter
OM: organic matter
CP: crude protein
EE: ether extract/crude fat
CF: crude fiber
HPLC: high-performance liquid chromatography
ATTDC: apparent total tract digestibility coefficients
TFC: total fecal collection method
SEM: standard error of the mean
D: diet
M: method
D × M: interaction between diets and methods.
